# Can Conization Specimens Predict Sentinel Lymph Node Status in Early-Stage Cervical Cancer? A SENTICOL Group Study

**DOI:** 10.3390/cancers13215423

**Published:** 2021-10-29

**Authors:** Vincent Balaya, Benedetta Guani, Julie Mereaux, Laurent Magaud, Basile Pache, Hélène Bonsang-Kitzis, Charlotte Ngô, David Desseauve, Patrice Mathevet, Fabrice Lécuru, on behalf of the SENTICOL Group

**Affiliations:** 1 Gynecology Department, Centre Hospitalo-Universitaire Vaudois, 1011 Lausanne, Switzerland; Benedetta.Guani@chuv.ch (B.G.); Basile.Pache@chuv.ch (B.P.); David.Desseauve@chuv.ch (D.D.); Patrice.Mathevet@chuv.ch (P.M.); 2 Department of Gynecology and Obstetrics, Foch Hospital, 92150 Suresnes, France; 3 Faculty of Biology and Medicine, University of Lausanne, 1015 Lausanne, Switzerland; 4 Department of Obstetrics and Gynecology, Cantonal Hospital, 1708 Fribourg, Switzerland; 5 Breast, Gynecology and Reconstructive Surgery Unit, Curie Institute, 75005 Paris, France; julie_mereaux@hotmail.fr (J.M.); fabrice.lecuru@curie.fr (F.L.); 6 Clinical Research and Epidemiology Department, Public Health Center, Hospices Civils de Lyon, F-69003 Lyon, France; laurent.magaud@chu-lyon.fr; 7 Gynecological and Breast Surgery and Cancerology Center, RAMSAY-Générale de Santé, Hôpital Privé des Peupliers, 75013 Paris, France; h.kitzisbonsang@yahoo.fr (H.B.-K.); charlotte.ngo2@gmail.com (C.N.)

**Keywords:** cervical cancer, sentinel lymph node, conization, SENTICOL, stromal invasion, lymphovascular space invasion

## Abstract

**Simple Summary:**

Lymph node involvement is one of the major prognosis factors for early-stage cervical cancer. Improvement in preoperative identification of node-positive patients may lead to a more accurate triage to primary chemoradiation for these patients instead of radical surgery followed by adjuvant radiotherapy, given the increased morbidity of combined treatment. Several studies have well established risk factors for node involvement, but they are based on final pathologic examination of radical hysterectomy specimens and are usually extrapolated for preoperative risk assessment. Among these risk factors, tumor size, lymphovascular space invasion (LVSI) and depth of stromal invasion might be assessed in conization specimens. Our findings suggest that patients with depth of stromal invasion lower than 10 mm and no LVSI in conization specimens had lower risk of micro- and macrometastatic SLN. In this subpopulation, full node dissection may be questionable in case of SLN unilateral detection.

**Abstract:**

Background: The prognosis of patients with cervical cancer is significantly worsened in case of lymph node involvement. The goal of this study was to determine whether pathologic features in conization specimens can predict the sentinel lymph node (SLN) status in early-stage cervical cancer. Methods: An ancillary analysis of two prospective multicentric database on SLN biopsy for cervical cancer (SENTICOL I and II) was carried out. Patients with IA to IB2 2018 FIGO stage, who underwent preoperative conization before SLN biopsy were included. Results: Between January 2005 and July 2012, 161 patients from 25 French centers fulfilled the inclusion criteria. Macrometastases, micrometastases and Isolated tumor cells (ITCs) were found in 4 (2.5%), 6 (3.7%) and 5 (3.1%) patients respectively. Compared to negative SLN patients, patients with micrometastatic and macrometastatic SLN were more likely to have lymphovascular space invasion (LVSI) (60% vs. 29.5%, *p* = 0.04) and deep stromal invasion (DSI) ≥ 10 mm (50% vs. 17.8%, *p* = 0.04). Among the 93 patients with DSI < 10 mm and absence of LVSI on conization specimens, three patients (3.2%) had ITCs and only one (1.1%) had micrometastases. Conclusions: Patients with DSI < 10 mm and no LVSI in conization specimens had lower risk of micro- and macrometastatic SLN. In this subpopulation, full node dissection may be questionable in case of SLN unilateral detection.

## 1. Introduction

Cervical cancer is the fourth most common cancer among women and the fourth leading cause of cancer-related deaths in females, with more than 600,000 newly diagnosed cases and 340,000 deaths each year [1]. The prognosis is significantly worsened in case of lymph node involvement [2], especially in case of paraaortic involvement [3], justifying its recent integration in the revised 2018 FIGO classification, which also added the possibility of preoperative lymph node staging by imaging [4]. Improvement in preoperative identification of node-positive patients may lead to a more accurate triage to primary chemoradiation for these patients instead of radical surgery followed by adjuvant radiotherapy, given the increased morbidity of combined treatment [5,6,7].

Several studies have well established risk factors for node involvement, such as tumor size larger than 20 mm, the presence of lymphovascular space invasion (LVSI), deeper stromal invasion and parametrial involvement [8,9,10,11,12]. Nonetheless, results of these studies are based on final pathologic examination of radical hysterectomy specimens and are usually extrapolated for preoperative risk assessment. As an alternative to pelvic lymphadenectomy, the feasibility and the reliability of SLN biopsy technique in early-stage cervical cancer have been widely described in the literature [13]. It has been demonstrated that bilateral negative SLN strongly predicted the absence of pelvic node involvement [14,15]. One of the main advantages of this technique is the ability to carry out SLN frozen-section analysis before performing radical hysterectomy. Node status may be known intraoperatively, and enhances the avoidance of inappropriate radical procedure instead of referring patients to concomitant chemoradiotherapy [7]. However, the clinical value of frozen section analysis remains questionable due to the high rate of false-negative results and requires a high level of expertise for the pathologists [16]. Another pitfall is SLN detection failure, which occurs bilaterally in 3.7–5.9% of cases and unilaterally in less than 15% of cases [17,18]. According to the MSKCC algorithm, full hemipelvic lymphadenectomy should be performed in cases of unilateral SLN failure detection [19]. However full lymph node dissection is associated with higher rate of surgical morbidity and worst quality of life [20,21].

Previous studies have investigated the predictive value of pathologic features of preoperative conization specimens for parametrial involvement [22], residual disease [23,24] and pathologic risk-factors [25] on radical hysterectomy specimens. Among the previously identified risk factors of lymph node metastasis, tumor size, LVSI and depth of stromal invasion might be assessed in conization specimens. Considering these factors before SLN mapping may help to adjust lymph node staging strategy.

The goal of this study was to determine whether pathological features in conization specimens can predict the sentinel lymph node (SLN) status in early-stage cervical cancer.

## 2. Materials and Methods

### 2.1. Population Study

An ancillary analysis of 2 prospective multicentric database on SLN biopsy for cervical cancer (SENTICOL I and II) was carried out. Design of both studies has already been reported elsewhere [14,20]. Succinctly, SENTICOL I aimed to assess the diagnostic value of SLN biopsy and SENTICOL II aimed to compare postoperative morbidity and quality of life after SLN biopsy alone versus SLN biopsy with pelvic lymphadenectomy. All patients included in both studies had early-stage cervical cancer smaller than 4 cm, no suspicious nodes at preoperative pelvic MRI, and underwent SLN mapping.

In the present study, patients with IA to IB2 2018 FIGO stage who underwent conization before SLN biopsy were included. Patients who had bilateral or unilateral SLN detection failure or preoperative brachytherapy were excluded. This study obtained approval from the Paris Descartes and Lyon Hospital Ethical Committees. Patients included in the two studies signed an informed consent stating the use of data for secondary analyses.

### 2.2. Data Analysis

All data were collected from two prospective multicentric databases. For each patient, demographic characteristics and clinical data were extracted and operative records were reviewed. Data about conization included histologic type, tumor size, depth of stromal invasion (DSI), the presence or not of LVSI and margin status. Conization was performed by cold-knife or loop electrical excision procedure depending on the inclusion center. All pathologic slides were analyzed at the center where they were performed by experienced gynecologic pathologists.

SLN detection was performed with a combined labeling technique (Patent blue and radioactive tracer). SLNs were analyzed after hematoxylineosin (HE) staining of 200-µm sections. All SLNs defined as negative by HE were submitted to ultrastaging protocol by using anti-cytokeratin antibodies (AE1-AE3 antibodies). Positive SLNs were classified according the tumor cells size: Isolated tumor cells (ITCs) were defined as <0.2 mm, micrometastases as between 0.2 and 2 mm, and macrometastases as >2 mm [26].

### 2.3. Statistical Analysis

Patients were divided into two groups according to the SLN status after ultrastaging: positive or negative. A first analysis was performed by considering all type of metastatic SLN (ITCs, micrometastases, and macrometastases). Since the presence of ITCs did not result in upstaging according to the 2018 revised FIGO classification, a second analysis was performed after excluding ITCs.

Qualitative variables were expressed as *n* (%) and were compared by applying the chi-square test (or Fisher’s test if the sample size was too small). Quantitative data were expressed as mean ± standard deviation (SD) and were compared by applying Student’s *t*-test. A univariate analysis was performed to identify clinicopathologic risk factors of SLN involvement in conization specimens. *p* values lower than 0.05 were retained as significance set. Receiver operating characteristic (ROC) analysis of the significant quantitative factors were made to define threshold values. Significant variables in univariate analysis were entered into a multivariate logistic regression model to determine preoperative variables independently associated with SLN involvement. Based on these independent variables, an asymptotic exact logistic regression test was applied to determine prediction risk of SLN involvement. All statistical analyses were carried out using XLStat Biomed software (AddInsoft V19.4, Paris, France).

## 3. Results

### 3.1. Clinicopathological Characteristics

Among the 405 patients enrolled in both studies between January 2005 and July 2012, 326 patients had successful bilateral SLN detected. Among them, preoperative conization was performed in 193 patients. After exclusion of 25 patients who had preoperative brachytherapy and 7 patients with missing data about conization specimens, 161 patients from 25 French centers were finally included for analysis. Clinical and surgicopathological features are presented in Table 1.

The median age was 39 years old [22–79 years], and the median body mass index (BMI) was 22.1 kg/m^2^ [15.6–42.2 kg/m^2^]. Most patients had IB1 and IB2 clinical 2018 FIGO stage (81.4%) and squamous cell carcinoma (76.3%).

The median number of SLNs harvested per patient was 3 [2,3,4,5,6,7,8]. The SLN mapping was mainly performed during the radical hysterectomy procedure (68.3%). In two cases, no surgery was performed after SLN biopsy, due to positive SLN at frozen section examination. After ultrastaging, macrometastases, micrometastases and isolated tumor cells (ITCs) were found in 4 (2.5%), 6 (3.7%) and 5 (3.1%) patients, respectively, whereas 146 patients (90.7%) had bilateral negative SLN. Compared to patients with clinical IB1 2018 FIGO stage, patients with IB2 had significantly more LVSI (41.2% vs. 21.9%, *p* < 0.0001) and more DSI ≥ 10 mm (47.1% vs. 21.1%, *p* = 0.001).

Of 161 patients who underwent conization, 48 had residual disease on final surgical specimens. Among the 118 patients who had tumor size lower than 20 mm on conization specimens, 11 patients (9.3%) had tumor size larger than 20 mm on final pathology. Among the 112 patients who did not have any LVSI on conization specimens, LVSI were finally found on surgical specimens in 10 patients (8.5%). Compared to final pathology, LVSI status assessment in conization specimens had a sensitivity of 84% (95%CI: [71.1–91.8%]), a specificity of 95.1% (95%CI: [88.7–98.1%]) and a negative predictive value of 92.4% (95%CI: [87.3–97.5%]). Among the 129 patients with DSI lower than 10 mm in conization specimens, 11 patients (8.5%) had DSI higher than 10 mm in surgical specimens.

### 3.2. Predictive Factors for Overall SLN Involvement

On the basis of univariate analysis, patients with positive SLN were more likely to have deeper stromal invasion (9.6 vs. 6.3 mm, *p* = 0.048) compared to negative SLN patients (Table 2). ROC analysis showed that a threshold of 10 mm for depth of stromal invasion in conization specimens predicted SLN involvement with a 42.9% sensitivity, 81.3% specificity and an area under the curve of 0.68, 95%IC = [0.52–0.84]. The rate of positive SLN patients were significantly higher in case of DSI > 10 mm (6/26) (23.1% vs. 7.5%, *p* = 0.04). The presence of LVSI was not significantly different between patients with negative SLN and those with positive SLNs. There were no significant differences in terms of histologic type, grade of differentiation, tumor size and resection margin status.

### 3.3. Predictive Factors for Micrometastatic and Macrometastic SLN Involvement

After excluding ITCs, univariate analysis revealed that patients with micrometastases and macrometastases had significantly deeper stromal invasion (10.3 mm vs. 6.3 mm, *p* = 0.04) and more LVSI (60% vs. 29.5%, *p* = 0.048) (Table 3). By multivariate analysis, a stromal invasion deeper than 10 mm in conization specimens was the unique independent factor for positive SLN (OR = 3.91, 95%CI = [1.03–14.9], *p* = 0.046).

Due to statistical significance, the DSI and the presence of LVSI on conization specimens were included to perform an asymptotic exact logistic regression test to determine prediction risk of micrometastatic or macrometastatic SLN (Table 4).

On the basis of these results, we suggested the following risk stratification of SLN involvement: patients with no risk factor may be defined as a low-risk group (90 patients—57.7%), patients with one risk factor (presence of LVSI or DSI ≥ 10 mm) an intermediate-risk group (52 patients—33.3%) and patients with two risk factors (presence of LVSI and DSI ≥ 10 mm) a high-risk group (14 patients—9.0%). The ROC curve using DSI and presence of LVSI in conization specimens for discriminating between low-risk, intermediate-risk and high-risk patients had an area under the curve of 0.693 (95%IC = [0.704–0.973], *p* < 0.0001). The overall SLN involvement rates for low-risk, intermediate-risk and high-risk groups were 4.3%, 17.3% and 25.0% respectively (*p* = 0.02). If ITCs were excluded, SLN involvement rates (micrometastases and macrometastases only) were 1.1% for low-risk group, 11.5% for intermediate-risk group and 21.4% for high-risk group (*p* = 0.009).

Among the whole cohort of 161 patients, 93 patients would be considered as low risk for SLN involvement, including 3 patients (3.2%) with ITCs and only one (1.1%) with micrometastases.

## 4. Discussion

Preoperative lymph node staging is of paramount importance in determining the most appropriate therapeutic strategy in cervical cancer. According to the 2018 FIGO classification, retroperitoneal lymph nodes could be assessed by imaging from stages I to III [4]. In a meta-analysis of 15 studies including 997 patients with cervical cancer, Xiao et al. have shown that conventional MRI had mild performance for assessing lymph node metastasis with a pooled sensitivity of 51% (95%CI: [42–60%]) and a pooled specificity of 90% (95%CI: [85–92%]) [27]. However, MRI performance may be improved by considering other clinicopathologic criteria. Ferrandina et al. defined “very low risk” patients for lymph node metastasis by the following criteria: preoperative negative pelvic lymph node status at MRI, tumor size < 20 mm, and squamous or adenosquamous histologic type [28,29]. In a cohort of 463 patients, the authors reported no case of lymph node metastases among the 161 patients who met these criteria [29]. The ability of MRI to predict lymph node metastases may also be improved by integrating radiomics features extracted from T2-weighted MRI and diffusion-weighted imaging with clinicopathologic risk-factors into a specific nomogram [30]. Currently, PET/CT is the imaging modality which shows the best diagnostic performance for detecting lymph node involvement in cervical cancer [31]. In a meta-analysis, Choi et al. found a pooled sensitivity and specificity of 82% and 95% for Positron emission tomography (PET) or PET/CT in identifying node positive patients [32]. Kim et al. suggested a nomogram incorporating both MRI and PET/CT features [33]. In addition to imaging, some authors described predictive models of lymph node assessment by taking into account some biomarkers [34,35]. Nonetheless, in early-stage cervical cancer, metastatic nodes are usually smaller than 2 mm up to 60% of positive node patients [36,37,38]. In this setting, pelvic MRI and PET-CT may fail to identify positive-node patients [39] since that the majority of metastatic nodes measured less than 10 mm [40].

Previous studies have investigated the predictive value of pathologic features of conization specimens [22,23,24,25] and highlighted that conization specimens should be considered in the preoperative staging of patients with early-stage cervical cancer. Smith et al. showed that the presence of LVSI in conization specimens and positive pelvic lymph nodes were predictive for parametrial involvement and radicality of hysterectomy could be determined based on these features [22]. Margin status of conization specimens may predict the presence of residual disease in radical hysterectomy specimens [23,24,25,41]. Holcomb et al. emphasized that the presence of LVSI and DSI were independent predictors of the depth of residual invasion in hysterectomy specimens [42]. The conization specimens may also provide information which could guide therapeutic strategy. Boren et al. found that presence of LVSI, positive conization margins, and endocervical curettage were associated with the use of adjuvant chemoradiation [43]. Hutchcraft et al. highlighted that LVSI in conization specimens was associated with intermediate-risk and high-risk pathologic criteria in hysterectomy specimens in 60% and 37% of cases, respectively [25]. Given that patients with intermediate or high risk criteria would be eligible for adjuvant therapy, LVSI status in conization specimens may influence the type of primary treatment [44]. Furthermore, several studies demonstrated that preoperative conization was associated with better oncologic outcomes [45,46,47]. This lower risk of recurrence associated with prior conization may be explained by decreased risk of tumor dissemination, especially in cases of negative margin status. In the current study, our results support the concept that conization pathologic features may help to assess SLN involvement risk. Patients with no LVSI and DSI < 10 mm on conization specimens might be considered as low risk of micrometastatic and macrometastatic SLN.

The presence of LVSI is one of the main risk factors for lymph node involvement [9,29], and has a prognostic impact on recurrence rate in early-stage cervical cancer [48,49]. In this study, we highlighted that the presence of LVSI in conization specimens was associated with micro- and macrometastatic SLN. This finding was similar to those in previously published studies [25,43]. Nonetheless, the correlation between the LVSI detected in biopsy or conization specimens and those found in hysterectomy specimens is still subject to debate [25,41,44,50,51]. In our study, LVSI status assessment in conization specimens had a negative predictive value of 92.4% (95%CI: [87.3–97.5%]), which was similar to values reported in the literature [41,44]. Bidus et al. reported that cold-knife conization and LEEP (Loop Electrical Excision Procedure) had a sensitivity of 37.5% and 50% respectively and a negative predictive value of 88% and 80% respectively [44]. By contrast, Boren et al. found a higher rate of LVSI in cone specimens (43%) than in hysterectomy specimens (20%) [43]. In a cohort of 297 conizations, Bai et al. reported a sensitivity and a negative predictive value of 70.5% and 89.5% of conization specimens with invaded margin for predicting LVSI in the final radical hysterectomy pathologic examination [41].

The patterns of lymphatic dissemination of cervical cancer from the cervix to pelvic nodes through the parametrium and the prognostic impact of depth of stromal invasion have already been demonstrated [52,53,54,55]. In a cohort of 375 patients, Kim et al. found that lymph node involvement was 3.6% in patients with DSI < 5 mm on hysterectomy specimens, whereas this rate increased to 23% in patients with DSI > 5 mm (*p* < 0.001) [51]. In a cohort of 496 patients, Nanthamongkolkul et al. found that deep stromal invasion in radical hysterectomy specimens was an independent factor of lymph node metastasis (OR = 3.5, 95%CI = [1.4–9.1], *p* = 0.01) [8]. Zhu et al. subdivided 3298 patients according to the ratio of DSI compared to the cervical wall thickness on hysterectomy specimens and they reported rates of lymph node involvement of 24.5% in case of DSI inner full-thickness, 42.8% in case of DSI equal to full-thickness and 66.3% in case of DSI outer full-thickness (*p* < 0.01) [12]. These findings are concordant with our results, which revealed that DSI > 10 mm measured in conization specimens was independently associated with the risk of micrometastatic and macrometastatic SLN. Although DSI in conization specimens seemed to be correlated with SLN status, this criterion is not predictive for pathologic risk factors on radical hysterectomy specimens [25]. Nonetheless, measurement of stromal invasion may lack reproducibility whether it is assessed by absolute size in mm [4,51], by third (less than 1/3 or 2/3) [12,54] or subjectively (superficial versus deep) [8]. While there was correlation with LVSI status and DSI to positive SLN, our results did not indicate any association between tumor size in conization specimens and the risk of micrometastatic/macrometastatic SLN. Since 73.3% of patients of our cohort had tumor size < 20 mm, we speculate that patients undergoing conization were more likely to have small or unseen tumors whereas patients with bulky tumors would require simple biopsy only. Nonetheless, in our cohort, some patients with bulky or visible tumors, underwent conization if initial cervical biopsy was not contributive (necrotic tumor) or physical examination under general anesthesia was required. This bias selection might explain this finding, as suggested by other authors [22,25,56]. Moreover, the measurement of cervical tumor size remains a hot topic [57]. Preoperatively, tumor size may be assessed by clinical palpation, during colposcopic examination, or by imaging such as MRI or ultrasound. Each modality has inherent limitations, such as observer subjectivity, tumor topography (exophytic and/or endocervical) or tumor shrinkage and therefore variable efficiency [58,59,60]. In addition, tumor size described at pathologic examination may differ whether it refers to “diameter” or “dimension”, and in which axis the measurement is performed [57]. This is particularly important, since discordance between clinical and pathologic tumor size may result in 12% of upstaging rate according to Vetter et al. [56]. The authors found that patients with small tumor < 20 mm and those undergoing preoperative conization had lower risks of upstaging.

A few limitations need to be noted regarding the current study. First, this is a retrospective analysis of two databases that were not designed with the scope of our study in mind, and some pathologic details could have been missed even if the data were prospectively collected. The method used for conization was not reported, whether it was performed by cold-knife or by loop electrical excision procedure. All pathologic slides were analyzed at the center where they were performed and were not submitted to central reviewing, thus leading to non-consensual reporting or heterogenous measurement methods. However, pathologic examination was carried out by experienced gynecologic pathologists and reports were discussed at a multidisciplinary tumor board. The small sample size of the data set limited the possibility of conducting thorough statistical analysis, resulting in a loss of power. This inconsistency may explain why this study failed to identify specific risk factors for ITCs. Nonetheless, the presence of ITCs is not involved in upstaging according to the revised 2018 FIGO classification [4], with their clinical impact remaining controversial [38,61]. Further data collection is required to determine exactly predictive pathologic features for such metastatic nodes. The generalizability of these results is subject to certain limitations in regard with the population study characteristics. For instance, our cohort was pre-screened due to restrictive inclusion criteria. Patients with locally advanced cervical cancer, bulky tumor > 40 mm or positive nodes at preoperative imaging were excluded from both SENTICOL I and SENTICOL II studies. As previously stated, a possible selection bias must be undertaken, since most of patients had tumor size smaller than 20 mm.

Notwithstanding these limitations, this study offers some insight into the implications of pathologic features of conization specimens for preoperative lymph node staging. Our study supports the idea that full hemipelvic node dissection may be questionable in the case of unilateral SLN detection failure for the patients considered as low risk of micro- and macrometastatic SLN based on conization features. By contrast, in the case of positive LVSI and/or DSI > 10 mm, accurate frozen section examination of SLN should be carefully carried out before performing radical hysterectomy due to high risk of micro- and macrometastatic SLN. This approach may result in better triage of patients at high risk of multimodality treatment.

## 5. Conclusions

In conclusion, patients with DSI < 10 mm and no LVSI in conization specimens had lower risk of micro- and macrometastatic SLN. In this subpopulation, full node dissection may be questionable in the case of SLN unilateral detection. By contrast, SLN mapping should be performed meticulously to avoid missing metastatic nodes in patients with DSI ≥ 10 mm and positive LVSI in conization specimens. Further studies with larger cohort of patients are required to confirm these findings.

## Figures and Tables

**Table 1:** Population characteristics.

Predictive Variable	Total Population *n* = 161	
	*n* Mean ± SD	[%] [Range]
Age [yrs]		
Mean	40.9 ± 10.5	[22–79]
BMI [kg/m^2^]		
Mean	23.3 ± 4.9	[15.6–42.2]
<18.5	12	7.5
18.5–25	111	68.9
<25–30	21	13.0
>30	17	10.6
Parity status		
0	46	28.6
≥1	125	71.4
Histology		
Squamous cell carcinoma	123	76.3
Adenocarcinoma	36	22.5
Other type	2	1.3
Grade of differenciation		
G1	56	52.3
G2	37	34.6
G3	14	13.1
Not specified	54	
Clinical 2018 FIGO stage		
IA1 with LVSI	11	6.8
IA2	19	11.8
IB1	114	70.8
IB2	17	10.6
Conization specimens pathologic examination		
***Tumor size***		
Mean (mm)	13.1 ± 7.7	[1–40]
<20 mm	118	73.3
≥20 mm	43	26.7
***Depth of stromal invasion***		
Mean (mm)	6.6 ± 6.0	[0–40]
<10 mm	129	80.1
≥10 mm	32	19.9
***LVSI***		
Yes	49	30.4
No	112	69.6
***Margin status***		
Positive	58	48.7
Negative	79	51.3
Not specified	24	
Surgery		
***Type of approach***		
Minimally invasive	150	93.2
Open	11	6.8
***Type of surgery***		
Radical hysterectomy	110	72.4
Radical trachelectomy	30	19.7
Simple hysterectomy	6	3.9
Simple trachelectomy	3	2.0
Not performed	2	1.3
Not specified	9	
SLN mapping		
Median number of SLN harvested per patient	3	[2–8]
***SLN status***		
Negative	146	90.7
ITCs	5	3.1
Micrometastases	6	3.7
Macrometastases	4	2.5
Final surgical specimens pathologic examination		
***Residual disease***		
Yes	48	33.3
No	96	66.7
Not specified	17	
***Tumor size***		
Median (mm)	12	[1–50]
<20 mm	30/48	62.5
≥20 mm	18/48	37.5
***Depth of stromal invasion***		
Mean (mm)	6.5	[0–32]
<10 mm	29/48	60.4
≥10 mm	19/48	39.6
***LVSI***		
Yes	50	32.9
No	102	67.1
Not specified	9	
***Margin status***		
Positive	4	2.5
Negative	155	97.5
Not specified	2	

BMI: Body mass index. FIGO: International Federation of Gynecology and Obstetrics. G: Grade.

**Table 2:** Predictive factors of SLN status on conization specimens (overall metastatic SLN type).

Predictive Variable	Patients with SLN − *n* = 146		Patients with SLN + *n* = 15		*p*
	*n* Mean ± SD	[%] [Range]	*n* Mean ± SD	[%] [Range]	
Age [yrs]					
Mean	41.0 ± 10.2	[22–79]	39.9 ± 13.7	[25–77]	*0.71*
BMI [kg/m^2^]					
Mean	23.3 ± 5.0	[15.6–42.2]	23.8 ± 4.6	[18.7–33.7]	*0.74*
Parity status					
0	41	28.1	5	33.3	*0.67*
≥1	105	71.9	10	66.7	
Histology					
Squamous cell carcinoma	112	76.6	11	73.3	*0.84*
Adenocarcinoma	32	22.1	4	26.7	
Other type	2	1.4	0	0.0	
Grade of differenciation					
G1	52	54.2	4	36.4	*0.34*
G2	31	32.3	6	54.5	
G3	13	13.5	1	9.1	
Not specified	50		4		
Clinical 2018 FIGO stage					
IA1 with LVSI	11	7.5	0	0.0	*0.14*
IA2	17	11.6	2	13.3	
IB1	105	71.9	9	60.0	
IB2	13	8.9	4	26.7	
Conization specimens pathologic examination					
***Tumor size***					
Mean (mm)	13.0 ± 7.7	[1–40]	15.1 ± 7.8	[4–30]	*0.31*
<20 mm	107	73.3	11	73.3	*0.99*
≥20 mm	39	26.7	4	26.7	
***Depth of stromal invasion***					
Mean (mm)	6.3 ± 6.0	[0–40]	9.6 ± 7.0	[0–23]	*0.049*
<10 mm	120	82.2	9	60.0	*0.04*
≥10 mm	26	17.8	6	40.0	
***LVSI***					
Yes	43	29.5	6	40.0	*0.40*
No	103	70.5	9	60.0	
***Margin status***					
Positive	52	41.6	6	50.0	*0.57*
Negative	73	58.4	6	50.0	
Not specified	21		3		

**Table 3:** Predictive factors of SLN status on conization specimens (micrometastases and macrometastases only).

Predictive Variable	Patients with SLN − *n* = 146		Patients with MIC or MAC *n* = 10		*p*
	*n* Mean ± SD	[%] [Range]	*n* Mean ± SD	[%] [Range]	
Age [yrs]					
Mean	41.0 ± 10.2	[22–79]	37.4 ± 10.2	[25–54]	*0.28*
BMI [kg/m^2^]					
Mean	23.3 ± 5.0	[15.6–42.2]	23.1 ± 5.5	[18.7–33.7]	*0.88*
Parity status					
0	41	28.1	3	30.0	*0.99*
≥1	105	71.9	7	70.0	
Histology					
Squamous cell carcinoma	112	76.6	8	80.0	*0.99*
Adenocarcinoma	32	22.1	2	20.0	
Other type	2	1.4	0	0.0	
Grade of differenciation					
G1	52	54.2	3	37.5	*0.68*
G2	31	32.3	4	50.0	
G3	13	13.5	1	12.5	
Not specified	50		2		
Clinical 2018 FIGO stage					
IA1 with LVSI	11	7.5	0	0.0	*0.58*
IA2	17	11.6	1	10.0	
IB1	105	71.9	7	70.0	
IB2	13	8.9	2	20.0	
Conization specimens pathologic examination					
***Tumor size***					
Mean (mm)	13.0 ± 7.7	[1–40]	15.5 ± 8.3	[4–30]	*0.32*
<20 mm	107	73.3	7	70.0	*0.73*
≥20 mm	39	26.7	3	30.0	
***Depth of stromal invasion***					
Mean (mm)	6.3 ± 6.0	[0–40]	10.3 ± 5.9	[4–20]	*0.04*
<10 mm	120	82.2	5	50.0	*0.03*
≥10 mm	26	17.8	5	50.0	
***LVSI***					
Yes	43	29.5	6	60.0	*0.048*
No	103	70.5	4	40.0	
***Margin status***					
Positive	52	41.6	6	66.7	*0.17*
Negative	73	58.4	3	33.3	
Not specified	21		1		

**Table 4:** SLN status probability according to DSI and LVSI on conization specimens.

	DSI	Presence of LVSI	Risk of SLN + (%)	95% CI	No. Total Patients	No. Patients with SLN+		% of Patients with SLN+
						**MIC**	**MAC**	
Low-risk	<10 mm	No	2.6	0.9–7.8	90	1	0	1.1
Intermediate-risk	<10 mm	Yes	7.5	2.6–19.8	35	21	2	11.4
	≥10 mm	No	9.6	2.8–27.9	17	1	1	11.7
High-risk	≥10 mm	Yes	24.1	9.6–48.6	14	2	1	21.4

## Data Availability

The data presented in this study are available on request from the corresponding author. The data are not publicly available due to privacy.
